# *N*-(3,4-Difluoro­phen­yl)-3,4,5-trimethoxy­benzamide

**DOI:** 10.1107/S1600536810013796

**Published:** 2010-04-24

**Authors:** Hyeong Choi, Byung Hee Han, Taewoo Lee, Sung Kwon Kang, Chang Keun Sung

**Affiliations:** a Department of Chemistry, Chungnam National University, Daejeon 305-764, Republic of Korea; b Department of Food Science and Technology, Chungnam National University, Daejeon 305-764, Republic of Korea

## Abstract

In the title amide, C_16_H_15_F_2_NO_4_, the dihedral angle between the benzene rings is 2.33 (15)°. Mol­ecules are linked in the crystal structure by N—H⋯O hydrogen bonding involving N—H and C=O groups of the amide function, leading to a supra­molecular chain along [100].

## Related literature

For background to the development of potent inhibitory agents of tyrosinase and melanin formation as whitening agents, see: Cabanes *et al.* (1994 ▶); Dawley & Flurkey (1993 ▶); Ha *et al.* (2007 ▶); Hong *et al.* (2008 ▶); Kwak *et al.* (2010 ▶); Lee *et al.* (2007 ▶); Nerya *et al.* (2003 ▶); Park *et al.* (2010 ▶); Sung & Samyang Genex (2001 ▶); Yi *et al.* (2009 ▶, 2010 ▶).
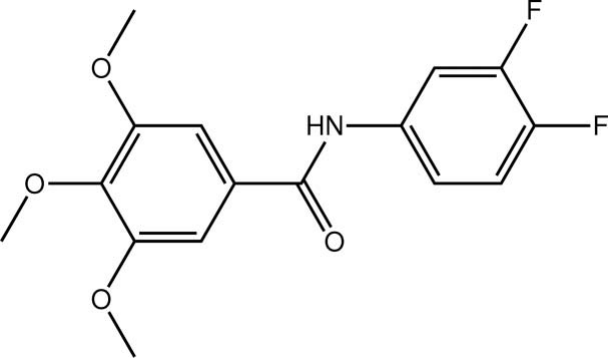


## Experimental

### 

#### Crystal data

C_16_H_15_F_2_NO_4_
*M* *_r_* = 323.29
Monoclinic, 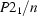

*a* = 5.0031 (3) Å
*b* = 8.8986 (5) Å
*c* = 32.726 (2) Å
β = 93.896 (4)°
*V* = 1453.59 (15) Å^3^
*Z* = 4
Mo *K*α radiation
μ = 0.12 mm^−1^
*T* = 174 K
0.12 × 0.05 × 0.04 mm

#### Data collection

Bruker SMART CCD area-detector diffractometer
10828 measured reflections
2634 independent reflections
1522 reflections with *I* > 2σ(*I*)
*R*_int_ = 0.080

#### Refinement

*R*[*F* ^2^ > 2σ(*F* ^2^)] = 0.065
*wR*(*F* ^2^) = 0.185
*S* = 1.05
2634 reflections
216 parameters
H atoms treated by a mixture of independent and constrained refinement
Δρ_max_ = 0.32 e Å^−3^
Δρ_min_ = −0.31 e Å^−3^

### 

Data collection: *SMART* (Bruker, 2002 ▶); cell refinement: *SAINT* (Bruker, 2002 ▶); data reduction: *SAINT*; program(s) used to solve structure: *SHELXS97* (Sheldrick, 2008 ▶); program(s) used to refine structure: *SHELXL97* (Sheldrick, 2008 ▶); molecular graphics: *ORTEP-3 for Windows* (Farrugia, 1997 ▶); software used to prepare material for publication: *WinGX* (Farrugia, 1999 ▶).

## Supplementary Material

Crystal structure: contains datablocks global, I. DOI: 10.1107/S1600536810013796/tk2658sup1.cif

Structure factors: contains datablocks I. DOI: 10.1107/S1600536810013796/tk2658Isup2.hkl

Additional supplementary materials:  crystallographic information; 3D view; checkCIF report

## Figures and Tables

**Table 1:** Hydrogen-bond geometry (Å, °)

*D*—H⋯*A*	*D*—H	H⋯*A*	*D*⋯*A*	*D*—H⋯*A*
N15—H15⋯O14^i^	0.93 (4)	2.02 (4)	2.872 (4)	152 (3)

Symmetry code: (i) 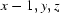.

**Fig. 1.**
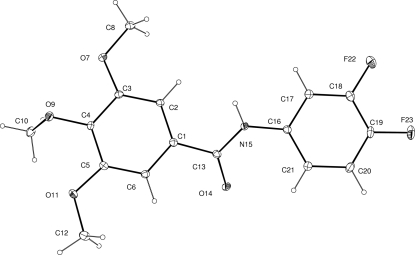
The molecular structure of (I), showing displacement ellipsoids drawn at the 30% probability level.

## supplementary crystallographic information

### Comment

Melanin synthesis is principally responsible for skin color and plays a key role in the prevention of UV-induced skin damages. Tyrosinase is the key enzyme (Ha *et al.*, 2007) that converts tyrosine to melanin and its inhibitors are target molecules for developing anti-pigmentation agents. Therefore, treatments using potent inhibitory agents on tyrosinase and melanin formation may be cosmetically useful. Common tyrosinase inhibitors (Dawley & Flurkey, 1993; Nerya *et al.*, 2003) are hydroquinone, ascorbic acid, kojic acid and arbutin (Cabanes *et al.*, 1994). Recently, a number of reports have focused on the development of new agents for the inhibition of tyrosinase. They contain aromatic, methoxy, hydroxyl (Hong *et al.*, 2008; Lee *et al.*, 2007), aldehyde (Yi *et al.*, 2010), amide (Kwak *et al.*, 2010), thiosemicarbazone (Yi *et al.*, 2009) groups in their respective molecule structure. The application of natural products as a melanin synthesis inhibitors has also attracted interest (Park *et al.*, 2010; Sung *et al.*, 2001). However, most of these are not sufficiently potent for practical use owing to their weak individual activities or due to safety concerns. Undoubtedly, significant research and development into novel tyrosinase inhibitors is required to generate molecules with better activities and reduced side-effects. In continuation of our program aimed to develop tyrosinase inhibitors, we have synthesized the title compound, *N*-(3,4-difluorophenyl)-3,4,5-trimethoxybenzamide, (I), from the reaction of 3,4-difluoroaniline with 3,4,5-trimethoxybenzoyl chloride under ambient condition. Herein, the crystal structure of (I) is described (Fig. 1).

The 3,4,5-trimethoxybenzoic acid moiety (except for the C10 methyl group) and 3,4-difluoroaniline group are essentially planar, with a mean deviations of 0.027 Å and 0.006 Å, respectively, from the corresponding least-squares plane defined by the ten and nine, respectively, constituent atoms. The dihedral angle between the benzene rings is 2.33 (15) °. The presence of intermolecular N15—H15···O14^i^ (symmetry code: (i) x-1, y, z) hydrogen bonds lead to the formation an 1-D supramolecular chain along the *a *axis, Table 1.

### Experimental

3,4,5-Trimethoxybenzoyl chloride and 3,4-difluoroaniline were purchased from Sigma Chemical Co. Solvents used for synthesis were redistilled before use. All other chemicals and solvents were of analytical grade and used without further purification. The title compound was prepared from the reaction of 3,4,5-trimethoxybenzoyl chloride (1.078 g, 5 mmol) and 3,4-difluoroaniline (0.5 g, 4 mmol) in THF with TEA (15 ml) as a catalyst. After being stirred for 5 h at 298 K, the mixture was treated with water and extracted with ethyl acetate. The combined extracts were dried over anhydrous magnesium sulfate. Removal of solvent gave a white solid (90%, m.pt. 428 K). Single crystals were obtained by slow evaporation of a methylene chloride and ethyl alcohol solution of (I) held at room temperature.

### Refinement

The amide-H atom was located in a difference Fourier map and refined freely. The remaining H atoms were positioned geometrically and refined using a riding model with C—H = 0.93-0.96 Å, and with U_iso_(H) = 1.2U_eq_ (carrier C) for aromatic and 1.5U_eq_(carrier C) for methyl H atoms.

### Crystal data

**Table:**

C_16_H_15_F_2_NO_4_	*F*(000) = 672
*M**_r_* = 323.29	*D*_x_ = 1.477 Mg m^−^^3^
Monoclinic, *P*2_1_/*n*	Mo *K*α radiation, λ = 0.71073 Å
Hall symbol: -P 2yn	Cell parameters from 761 reflections
*a* = 5.0031 (3) Å	θ = 2.6–19.9°
*b* = 8.8986 (5) Å	µ = 0.12 mm^−^^1^
*c* = 32.726 (2) Å	*T* = 174 K
β = 93.896 (4)°	Needle, colourless
*V* = 1453.59 (15) Å^3^	0.12 × 0.05 × 0.04 mm
*Z* = 4	

### Data collection

**Table:**

Bruker SMART CCD area-detector diffractometer	*R*_int_ = 0.080
φ and ω scans	θ_max_ = 25.5°, θ_min_ = 2.4°
10828 measured reflections	*h* = −4→6
2634 independent reflections	*k* = −10→6
1522 reflections with *I* > 2σ(*I*)	*l* = −39→34

### Refinement

**Table:**

Refinement on *F*^2^	H atoms treated by a mixture of independent and constrained refinement
Least-squares matrix: full	*w* = 1/[σ^2^(*F*_o_^2^) + (0.0682*P*)^2^ + 1.4724*P*] where *P* = (*F*_o_^2^ + 2*F*_c_^2^)/3
*R*[*F*^2^ > 2σ(*F*^2^)] = 0.065	(Δ/σ)_max_ < 0.001
*wR*(*F*^2^) = 0.185	Δρ_max_ = 0.32 e Å^−^^3^
*S* = 1.05	Δρ_min_ = −0.31 e Å^−^^3^
2634 reflections	Extinction correction: *SHELXL97* (Sheldrick, 2008), Fc^*^=kFc[1+0.001xFc^2^λ^3^/sin(2θ)]^-1/4^
216 parameters	Extinction coefficient: 0.014 (2)
0 restraints	

### Special details

**Table:**

Geometry. All esds (except the esd in the dihedral angle between two l.s. planes) are estimated using the full covariance matrix. The cell esds are taken into account individually in the estimation of esds in distances, angles and torsion angles; correlations between esds in cell parameters are only used when they are defined by crystal symmetry. An approximate (isotropic) treatment of cell esds is used for estimating esds involving l.s. planes.

### Fractional atomic coordinates and isotropic or equivalent isotropic displacement parameters (Å^2^)

**Table:**

	*x*	*y*	*z*	*U*_iso_*/*U*_eq_	
C1	0.6607 (7)	0.3453 (4)	0.11117 (12)	0.0287 (9)	
C2	0.4753 (7)	0.2630 (5)	0.13169 (11)	0.0295 (9)	
H2	0.3569	0.1988	0.1172	0.035*	
C3	0.4671 (7)	0.2766 (4)	0.17375 (12)	0.0290 (9)	
C4	0.6362 (7)	0.3786 (4)	0.19522 (11)	0.0269 (9)	
C5	0.8177 (7)	0.4651 (4)	0.17433 (12)	0.0308 (10)	
C6	0.8325 (7)	0.4471 (4)	0.13255 (12)	0.0297 (9)	
H6	0.9558	0.5022	0.1187	0.036*	
O7	0.3015 (5)	0.1970 (3)	0.19728 (8)	0.0361 (7)	
C8	0.1331 (8)	0.0851 (5)	0.17712 (13)	0.0387 (11)	
H8A	0.0082	0.1328	0.1577	0.058*	
H8B	0.0368	0.0323	0.1971	0.058*	
H8C	0.2416	0.0154	0.1632	0.058*	
O9	0.6159 (5)	0.3968 (3)	0.23691 (8)	0.0372 (8)	
C10	0.8440 (8)	0.3424 (5)	0.26132 (13)	0.0436 (12)	
H10A	0.8604	0.2359	0.2575	0.065*	
H10B	0.8218	0.3633	0.2897	0.065*	
H10C	1.0027	0.3915	0.2531	0.065*	
O11	0.9674 (5)	0.5622 (3)	0.19817 (8)	0.0402 (8)	
C12	1.1391 (8)	0.6627 (5)	0.17819 (14)	0.0445 (12)	
H12A	1.2818	0.6068	0.1672	0.067*	
H12B	1.2129	0.7351	0.1976	0.067*	
H12C	1.0381	0.7138	0.1564	0.067*	
C13	0.6990 (7)	0.3258 (4)	0.06673 (12)	0.0317 (10)	
O14	0.9186 (5)	0.3442 (3)	0.05305 (8)	0.0409 (8)	
N15	0.4757 (6)	0.2891 (4)	0.04286 (10)	0.0299 (8)	
H15	0.313 (8)	0.294 (4)	0.0547 (11)	0.029 (10)*	
C16	0.4673 (7)	0.2563 (5)	0.00065 (12)	0.0305 (9)	
C17	0.2768 (8)	0.1532 (5)	−0.01466 (13)	0.0389 (11)	
H17	0.1633	0.1069	0.0028	0.047*	
C18	0.2571 (9)	0.1203 (5)	−0.05565 (13)	0.0426 (11)	
C19	0.4247 (9)	0.1857 (5)	−0.08182 (12)	0.0415 (11)	
C20	0.6105 (9)	0.2881 (6)	−0.06747 (13)	0.0493 (13)	
H20	0.7228	0.3337	−0.0853	0.059*	
C21	0.6312 (8)	0.3240 (5)	−0.02595 (12)	0.0402 (11)	
H21	0.7571	0.3944	−0.0161	0.048*	
F22	0.0737 (6)	0.0196 (3)	−0.07070 (8)	0.0697 (9)	
F23	0.3998 (5)	0.1491 (3)	−0.12188 (7)	0.0625 (9)	

### Atomic displacement parameters (Å^2^)

**Table:**

	*U*^11^	*U*^22^	*U*^33^	*U*^12^	*U*^13^	*U*^23^
C1	0.0233 (19)	0.032 (2)	0.030 (2)	0.0051 (18)	−0.0008 (16)	−0.0001 (18)
C2	0.0215 (18)	0.035 (2)	0.032 (2)	−0.0027 (17)	0.0031 (16)	−0.0032 (18)
C3	0.0236 (19)	0.030 (2)	0.034 (2)	0.0022 (17)	0.0048 (17)	0.0008 (19)
C4	0.0277 (19)	0.032 (2)	0.021 (2)	0.0052 (18)	0.0021 (16)	−0.0014 (18)
C5	0.028 (2)	0.028 (2)	0.035 (3)	0.0000 (18)	−0.0016 (17)	−0.0020 (19)
C6	0.027 (2)	0.030 (2)	0.031 (2)	−0.0011 (18)	0.0014 (16)	0.0047 (18)
O7	0.0376 (15)	0.0399 (18)	0.0314 (16)	−0.0088 (14)	0.0071 (12)	0.0033 (13)
C8	0.039 (2)	0.033 (2)	0.045 (3)	−0.005 (2)	0.008 (2)	0.007 (2)
O9	0.0331 (15)	0.0477 (19)	0.0311 (17)	0.0038 (14)	0.0041 (12)	−0.0016 (14)
C10	0.037 (2)	0.060 (3)	0.034 (3)	0.008 (2)	0.0027 (19)	0.005 (2)
O11	0.0464 (17)	0.0383 (17)	0.0351 (18)	−0.0121 (14)	−0.0030 (13)	−0.0064 (14)
C12	0.042 (2)	0.040 (3)	0.051 (3)	−0.007 (2)	−0.004 (2)	0.001 (2)
C13	0.025 (2)	0.035 (2)	0.035 (2)	0.0000 (18)	0.0010 (17)	−0.0018 (19)
O14	0.0242 (14)	0.066 (2)	0.0332 (17)	−0.0050 (14)	0.0079 (12)	−0.0011 (15)
N15	0.0239 (17)	0.041 (2)	0.025 (2)	0.0001 (16)	0.0027 (14)	−0.0020 (16)
C16	0.0235 (19)	0.036 (2)	0.032 (2)	0.0010 (18)	0.0012 (16)	−0.0018 (19)
C17	0.038 (2)	0.044 (3)	0.034 (3)	−0.005 (2)	0.0042 (19)	−0.004 (2)
C18	0.046 (3)	0.039 (3)	0.041 (3)	0.000 (2)	−0.003 (2)	−0.008 (2)
C19	0.047 (3)	0.056 (3)	0.021 (2)	0.013 (2)	0.0002 (19)	−0.005 (2)
C20	0.044 (3)	0.074 (4)	0.031 (3)	−0.001 (3)	0.006 (2)	0.011 (3)
C21	0.038 (2)	0.051 (3)	0.032 (3)	−0.007 (2)	0.0018 (19)	0.001 (2)
F22	0.085 (2)	0.072 (2)	0.0507 (18)	−0.0236 (18)	−0.0041 (15)	−0.0209 (16)
F23	0.0721 (18)	0.087 (2)	0.0284 (16)	0.0145 (16)	0.0001 (13)	−0.0119 (14)

### Geometric parameters (Å, °)

**Table:**

C1—C2	1.390 (5)	O11—C12	1.429 (5)
C1—C6	1.402 (5)	C12—H12A	0.96
C1—C13	1.490 (5)	C12—H12B	0.96
C2—C3	1.385 (5)	C12—H12C	0.96
C2—H2	0.93	C13—O14	1.226 (4)
C3—O7	1.367 (4)	C13—N15	1.358 (5)
C3—C4	1.397 (5)	N15—C16	1.410 (5)
C4—O9	1.384 (4)	N15—H15	0.93 (4)
C4—C5	1.403 (5)	C16—C21	1.375 (5)
C5—O11	1.355 (4)	C16—C17	1.392 (5)
C5—C6	1.383 (5)	C17—C18	1.370 (6)
C6—H6	0.93	C17—H17	0.93
O7—C8	1.436 (5)	C18—F22	1.352 (5)
C8—H8A	0.96	C18—C19	1.368 (6)
C8—H8B	0.96	C19—F23	1.348 (5)
C8—H8C	0.96	C19—C20	1.362 (6)
O9—C10	1.432 (4)	C20—C21	1.393 (6)
C10—H10A	0.96	C20—H20	0.93
C10—H10B	0.96	C21—H21	0.93
C10—H10C	0.96		
			
C2—C1—C6	120.4 (4)	C5—O11—C12	117.5 (3)
C2—C1—C13	123.0 (4)	O11—C12—H12A	109.5
C6—C1—C13	116.6 (3)	O11—C12—H12B	109.5
C3—C2—C1	120.0 (4)	H12A—C12—H12B	109.5
C3—C2—H2	120	O11—C12—H12C	109.5
C1—C2—H2	120	H12A—C12—H12C	109.5
O7—C3—C2	125.1 (3)	H12B—C12—H12C	109.5
O7—C3—C4	115.0 (3)	O14—C13—N15	123.0 (4)
C2—C3—C4	119.9 (3)	O14—C13—C1	121.3 (3)
O9—C4—C3	119.3 (3)	N15—C13—C1	115.7 (3)
O9—C4—C5	120.6 (3)	C13—N15—C16	125.6 (3)
C3—C4—C5	120.1 (3)	C13—N15—H15	118 (2)
O11—C5—C6	125.3 (4)	C16—N15—H15	117 (2)
O11—C5—C4	114.8 (3)	C21—C16—C17	119.0 (4)
C6—C5—C4	119.8 (4)	C21—C16—N15	123.4 (4)
C5—C6—C1	119.7 (4)	C17—C16—N15	117.6 (3)
C5—C6—H6	120.1	C18—C17—C16	119.7 (4)
C1—C6—H6	120.1	C18—C17—H17	120.2
C3—O7—C8	117.4 (3)	C16—C17—H17	120.2
O7—C8—H8A	109.5	F22—C18—C19	118.9 (4)
O7—C8—H8B	109.5	F22—C18—C17	120.0 (4)
H8A—C8—H8B	109.5	C19—C18—C17	121.1 (4)
O7—C8—H8C	109.5	F23—C19—C20	120.8 (4)
H8A—C8—H8C	109.5	F23—C19—C18	119.1 (4)
H8B—C8—H8C	109.5	C20—C19—C18	120.1 (4)
C4—O9—C10	113.7 (3)	C19—C20—C21	119.6 (4)
O9—C10—H10A	109.5	C19—C20—H20	120.2
O9—C10—H10B	109.5	C21—C20—H20	120.2
H10A—C10—H10B	109.5	C16—C21—C20	120.6 (4)
O9—C10—H10C	109.5	C16—C21—H21	119.7
H10A—C10—H10C	109.5	C20—C21—H21	119.7
H10B—C10—H10C	109.5		

### Hydrogen-bond geometry (Å, °)

**Table:**

*D*—H···*A*	*D*—H	H···*A*	*D*···*A*	*D*—H···*A*
N15—H15···O14^i^	0.93 (4)	2.02 (4)	2.872 (4)	152 (3)

Symmetry codes: (i) *x*−1, *y*, *z*.
